# Supplementary material for: Defining the Basal and Immunomodulatory Mediator-Induced Phosphoprotein Signature in Pediatric B Cell Acute Lymphoblastic Leukemia (B-ALL) Diagnostic Samples
Source: Int J Mol Sci. 2023 Sep 11;24(18):13937. doi: 10.3390/ijms241813937 (PMC10531382; doi:10.3390/ijms241813937)
Supplement: Supplementary file 1 [file ijms-24-13937-s001.zip › Supplementary Legends.pdf]

Supplementary Figure Legends

Supplementary Figure S1. Gating strategy to identify relevant cellular subsets for phosflow and cell surface receptor expression analyses. Polychromatic flow cytometry and a hierarchical gating strategy was utilized to identify leukemic B-ALL blasts, non-malignant mature B cells and T cells in diagnostic specimens obtained from pediatric subjects with confirmed diagnosis of B-ALL (Panels a, b). Hematogones, mature B cells and mature T cells were identified in non-leukemic bone marrow specimens as depicted (Panel c). For cell surface receptor expression analyses, the MAb against cyCD79a was replaced by a MAb against CD19. *A MAb targeting CD34 was used in those cases where the leukemic blasts lacked CD10 expression. Downstream analyses of the phosphoprotein signature and receptor surface expression was performed by further sub-gating on the identified relevant cellular subsets.

Supplementary Figure S2. Comparison of basal hyperphosphorylation status of proximal signaling nodes for leukemic blasts and hematogones. The basal phosphorylation signal for the signaling nodes listed along the y-axes was measured following PBS treatment by phosflow (a-e). The phosphorylation signal data are depicted in the form of MFI for the blasts (BL; salmon-colored filled circles) identified in the diagnostic samples of confirmed cases of pediatric B-ALL (*n*=63), and hematogones (H; purple-colored filled circles) identified in non-leukemic bone marrow specimens (*n*=4-8). Statistically significant differences were identified based on the statistical tests applied as listed in the Materials and Methods. Individual data points are shown in addition to the mean±SEM. A p-value <0.05 is considered significant. Each study subject was evaluated once.

Supplementary Figure S3. Comparison of immunomodulatory mediator-induced phosphorylation of proximal signaling nodes for leukemic blasts (BL) and hematogones (H). The phosphorylation signal for the signaling nodes listed along the y-axes was measured by phosflow following treatment with the indicated immunomodulatory mediators (rhIFNγ ; rhIL-2; rhIL-10; rhIL-4; rhIL-7 rhIL-21 and trimerized CD40L) (a-g). The numerical values along the y-axes denote the fold-change (FC) in the MFI of the phosphorylation signal of the signaling node following treatment with the signaling input compared to PBS treatment which is normalized to one. Individual data points are shown in addition to the mean±SEM. Statistically significant differences were identified based on the statistical tests applied as listed in the Materials and Methods. A p-value < 0.05 is considered statistically significant. For the leukemic samples (salmon-colored filled circles), *n*=63 for IFNγ, IL-2, IL-4, IL-10 and CD40L; *n*=59 for IL-7, and *n*=43 for IL-21. For the non-leukemic samples (purple-colored filled circles), *n*=4-8. Each study subject was evaluated once.

Supplementary Figure S4. IL-2R, IL-4R and IL-21R surface expression observed for non-leukemic bone-marrow specimens. The background-subtracted frequencies (%) of hematogones (H), mature B cells (MB) and mature T cells (T) in non-leukemic bone-marrow specimens (*n*=4), expressing IL-2Rα (CD25) (a), IL-2Rβ (CD122) (b), IL-4Rα (CD124) (c) and IL-21R (CD360) (d), on their surface was assessed by flow cytometry. The background signal was determined by staining an aliquot of each specimen with dose-matched, isotype control antibodies. Individual data points are shown in addition to the mean±SEM. A p-value <0.05 is considered significant. Each study subject was evaluated once.

Supplementary Figure S5. Comparison of IFNγR1 (CD119) and IFNγR2 surface expression on leukemic blasts versus hematogones. The background-subtracted frequencies (%) of leukemic blasts (BL; *n*=8) and hematogones (H) (*n*=4) expressing IFNγR1 (CD119) (a) and IFNγR2 (b) on their surface was assessed by flow cytometry. The background signal was determined by staining an aliquot of each specimen with dose-matched, isotype control antibodies. Individual data points are shown in addition to the mean±SEM. A p-value <0.05 is considered significant. Each study subject was evaluated once.

Supplementary Figure S6. Measurement of IL-10Rα (CD210), IL-10Rβ and CD40 surface expression in non-leukemic bone-marrow specimens. The background-subtracted frequencies (%) of hematogones (H), mature B cells (MB) and mature T cells (T) in non-leukemic bone-marrow specimens (*n*=4-8), expressing IL-10Rα (CD210) (a), IL-10Rβ (c) and CD40 (e), on their surface was assessed by flow cytometry. The background-subtracted MFI of IL-10Rα (CD210) (b), IL-10Rβ (d), and CD40 (f), surface expression was also assessed. The background signal was determined by staining an aliquot of each specimen with dose-matched, isotype control antibodies. Individual data points are shown in addition to the mean±SEM. A p-value <0.05 is considered significant. Each study subject was evaluated once.

Supplementary Figure S7. Comparison of IL-21R MFI for leukemic specimens. The background subtracted MFI of IL-21R surface expression was evaluated for blasts (BL), mature B cells (MB) and mature T cells (T) in leukemic diagnostic specimens (*n*=8). The background signal was determined by staining an aliquot of each specimen with dose-matched, isotype control antibodies. Individual data points are shown in addition to the mean±SEM. A p-value <0.05 is considered significant. Each study subject was evaluated once.

Supplementary Tables

Supplementary Table S1: Study subject related clinical data obtained at the time of diagnosis.

Supplementary Table S2: Study subject related phosflow data based on the clinical subtypes used for the stratification of pediatric B-ALL patients at the time of diagnosis.
