# Supplementary figures and images for: Defining the Basal and Immunomodulatory Mediator-Induced Phosphoprotein Signature in Pediatric B Cell Acute Lymphoblastic Leukemia (B-ALL) Diagnostic Samples
Source: Int J Mol Sci. 2023 Sep 11;24(18):13937. doi: 10.3390/ijms241813937 (PMC10531382; doi:10.3390/ijms241813937)

# Supplementary Figure S1

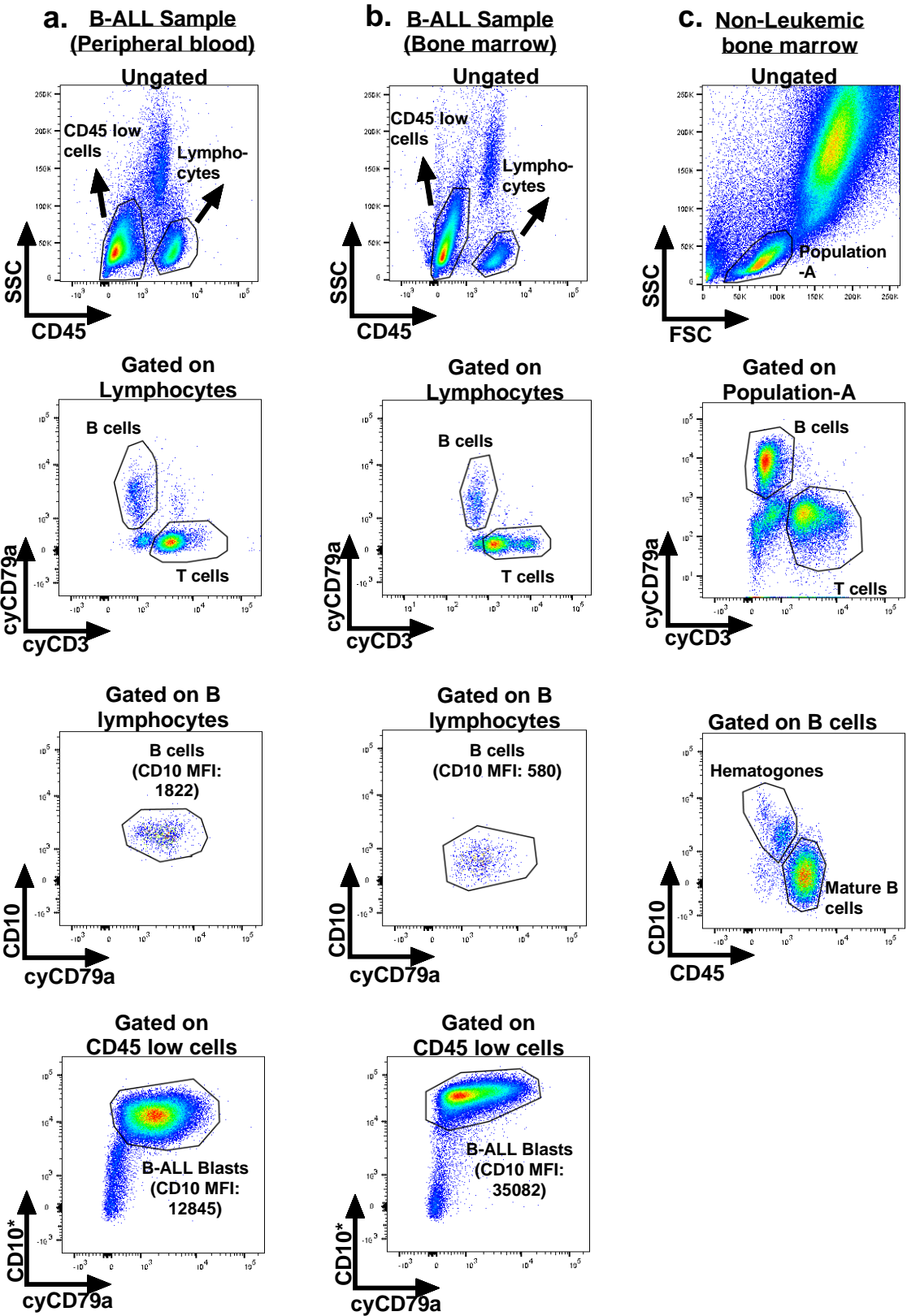

Supplement: Supplementary file 1 [file ijms-24-13937-s001.zip › Figure S1.pdf]

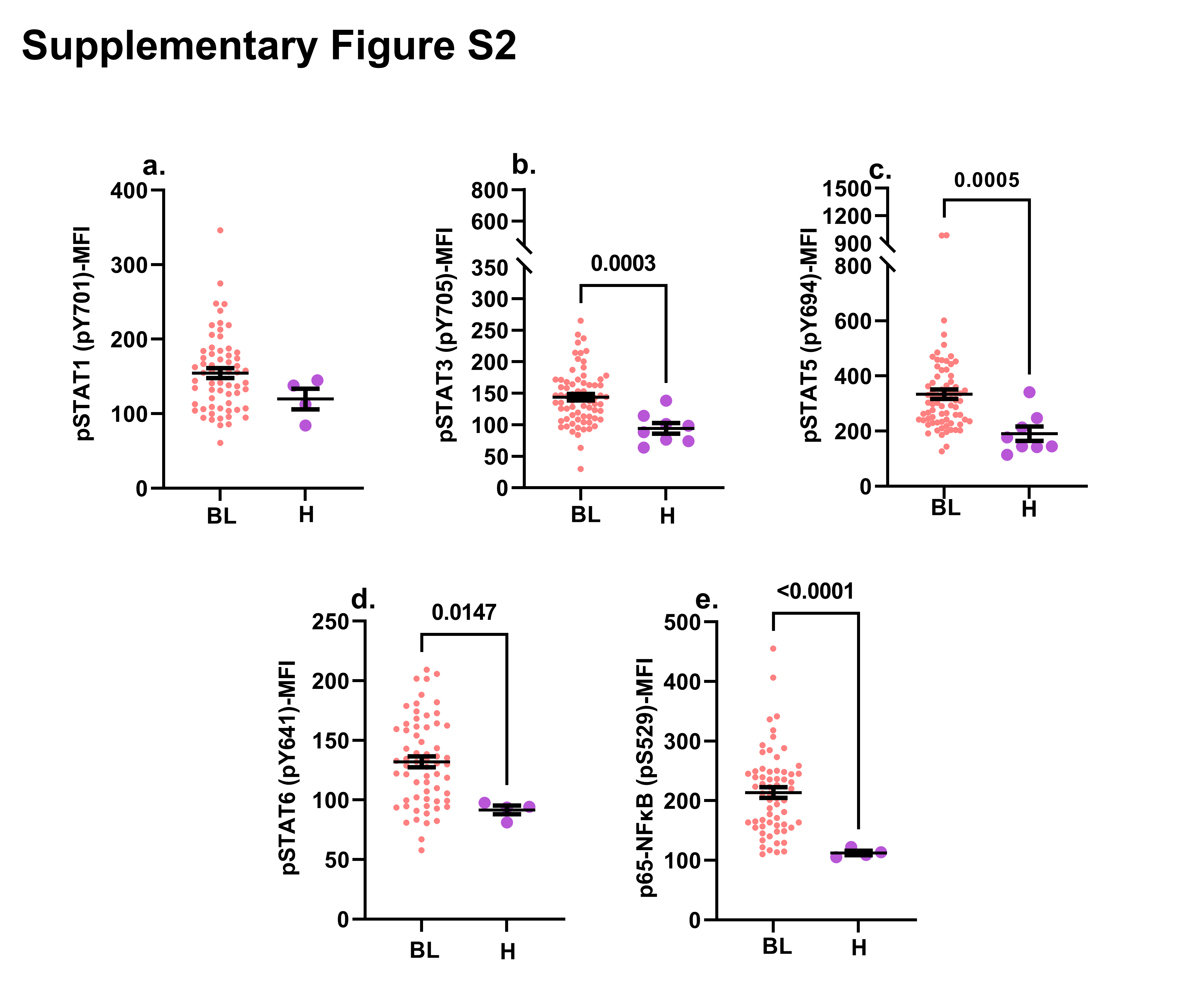

Supplement: Supplementary file 1 [file ijms-24-13937-s001.zip › Figure S2.tif]

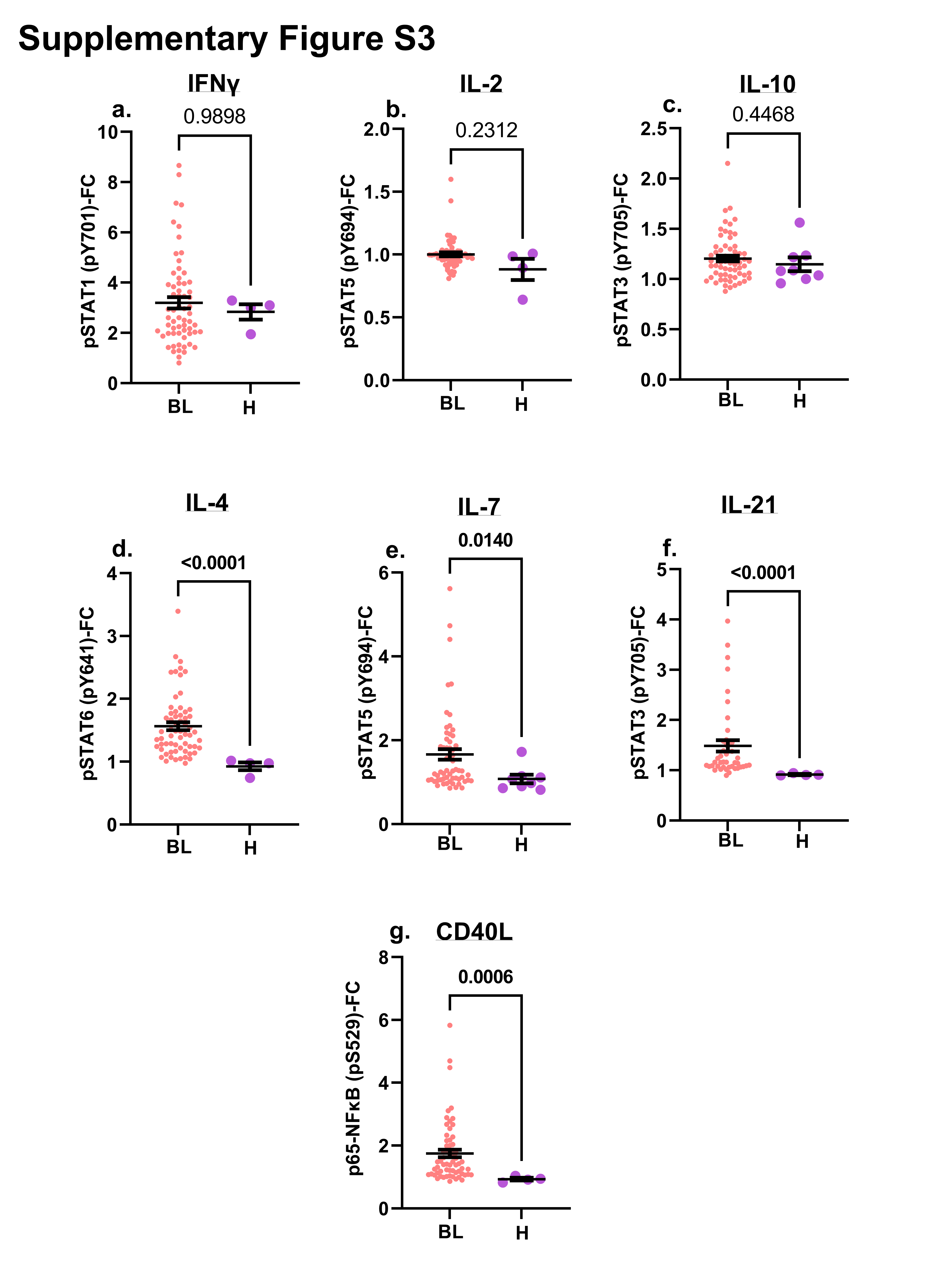

Supplement: Supplementary file 1 [file ijms-24-13937-s001.zip › Figure S3.tif]

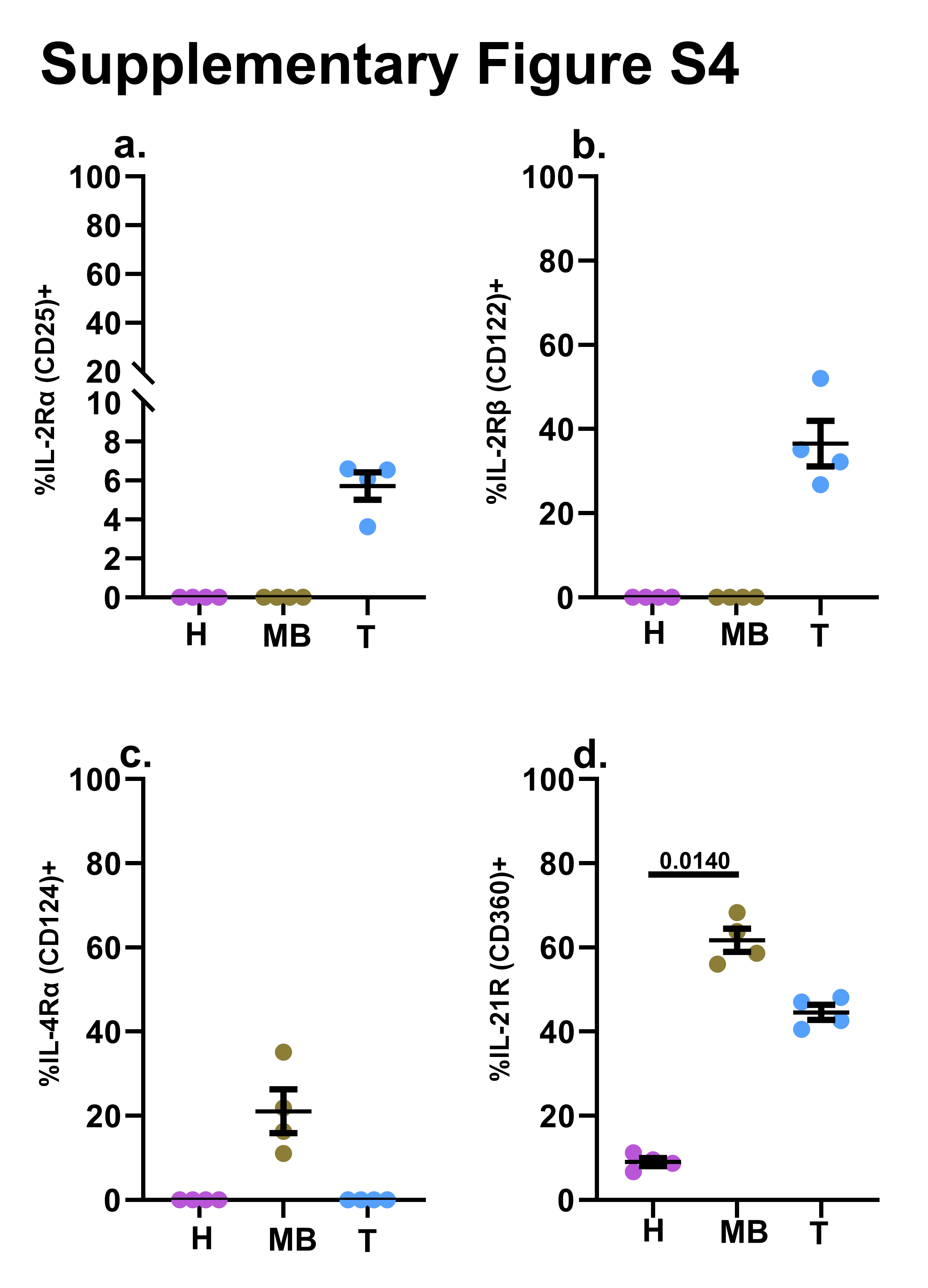

Supplement: Supplementary file 1 [file ijms-24-13937-s001.zip › Figure S4.tif]

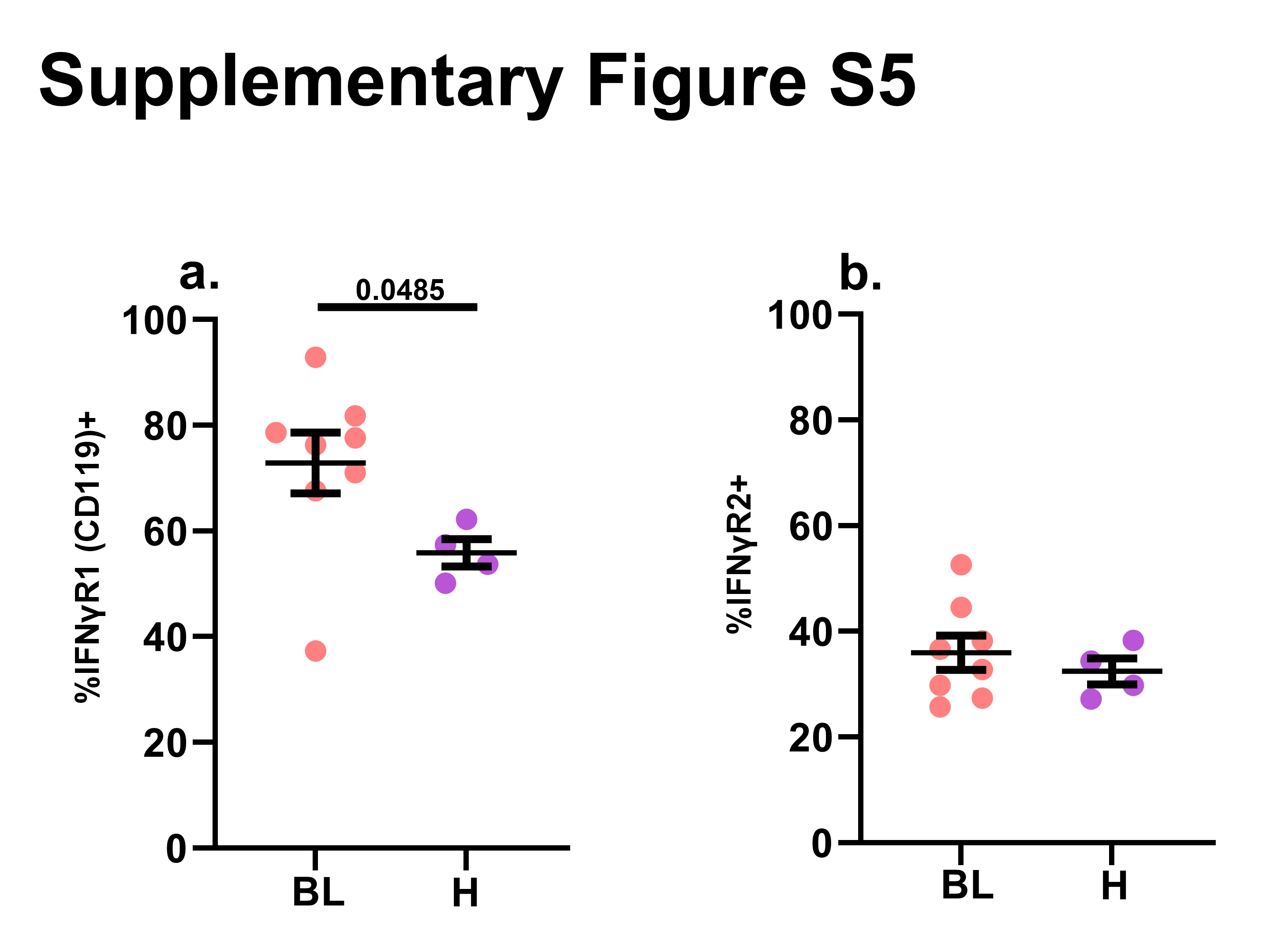

Supplement: Supplementary file 1 [file ijms-24-13937-s001.zip › Figure S5.tif]

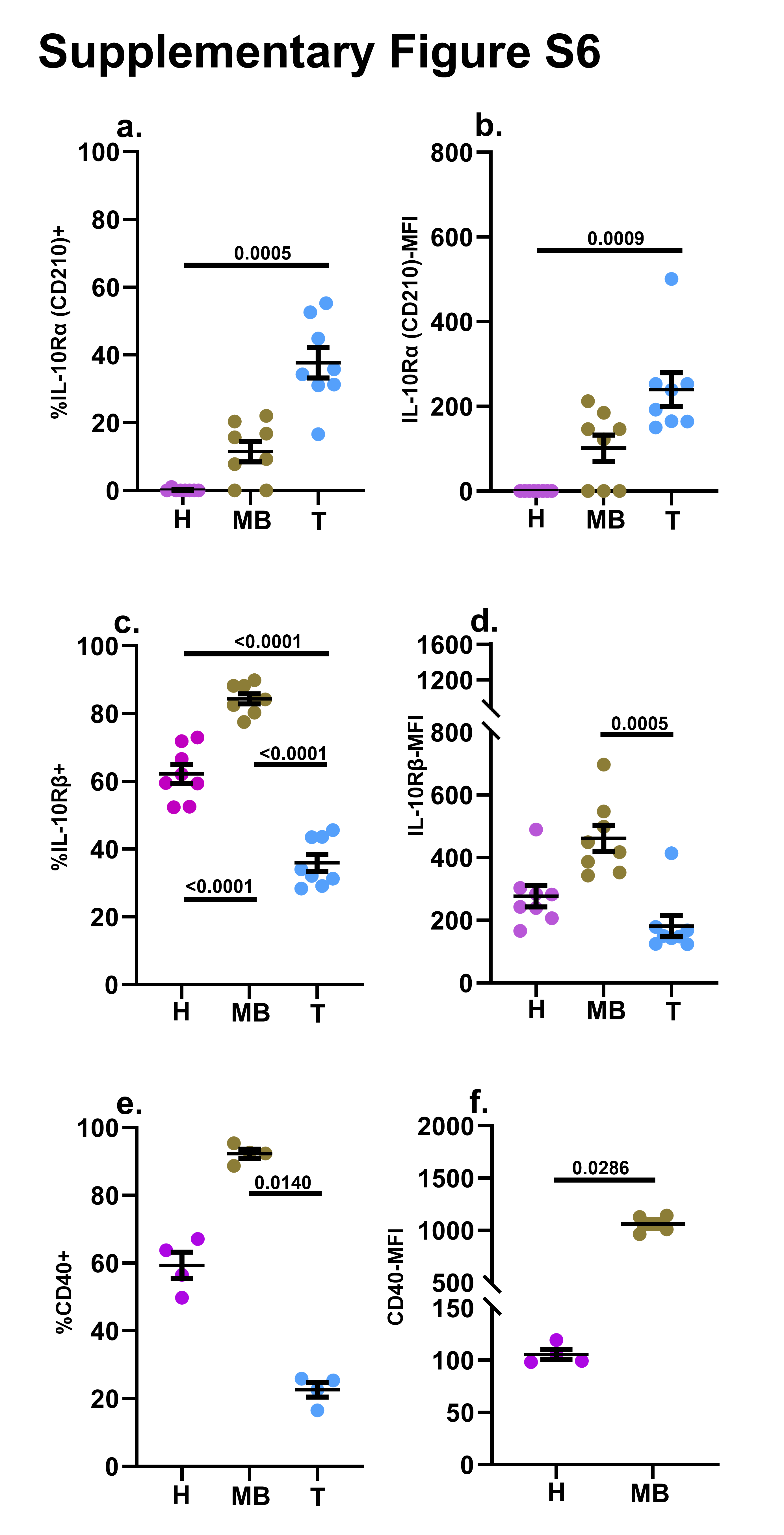

Supplement: Supplementary file 1 [file ijms-24-13937-s001.zip › Figure S6.tif]

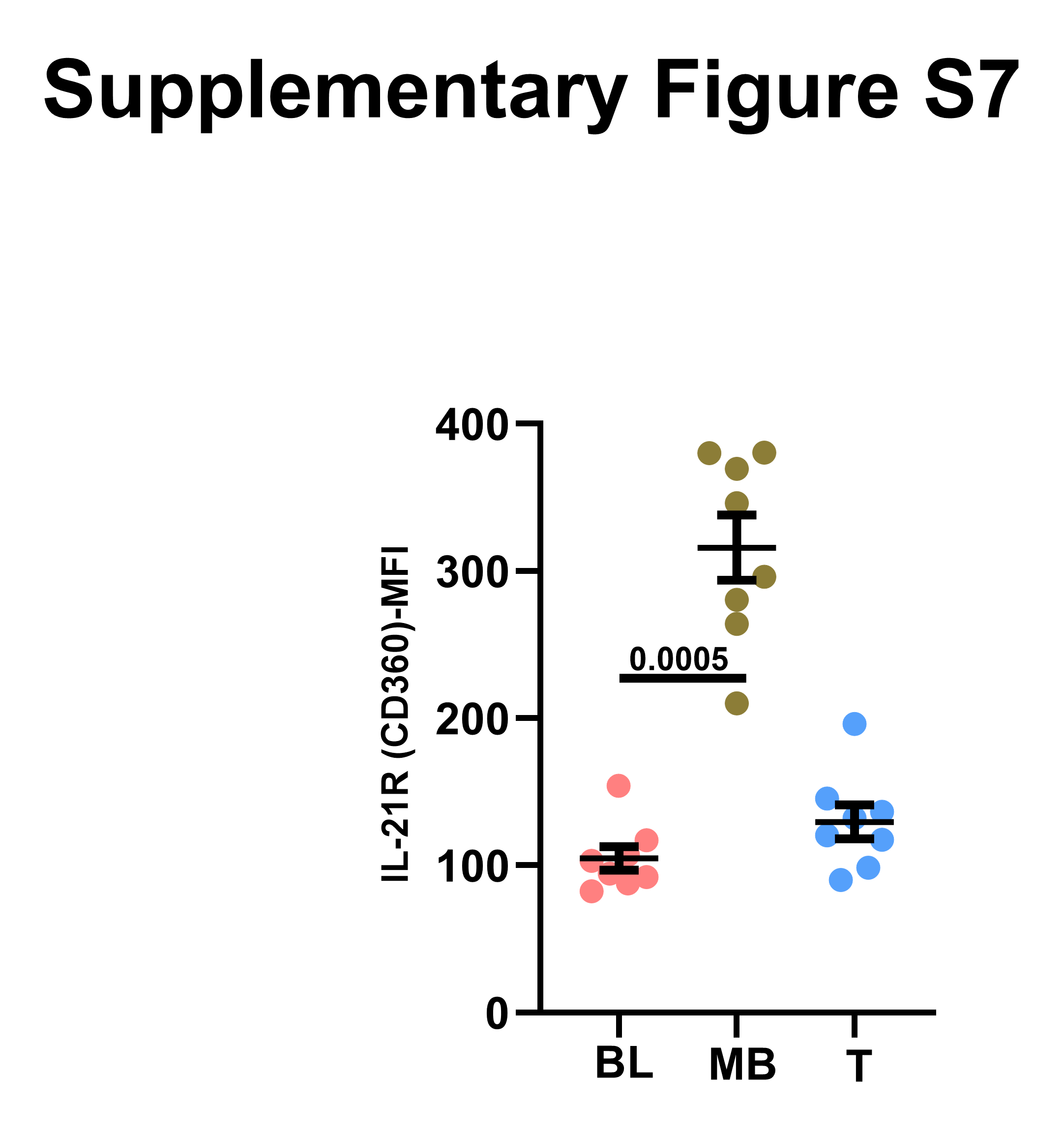

Supplement: Supplementary file 1 [file ijms-24-13937-s001.zip › Figure S7.tif]
